# Supplementary material for: Citric Acid-Mediated Microwave-Hydrothermal Synthesis of Mesoporous F-Doped HAp Nanorods from Bio-Waste for Biocidal Implant Applications
Source: Nanomaterials (Basel). 2022 Jan 19;12(3):315. doi: 10.3390/nano12030315 (PMC8840346; doi:10.3390/nano12030315)
Supplement: Supplementary file 1 [file nanomaterials-12-00315-s001.zip › nanomaterials-1505983-supplementary.pdf]

<u>**Supplementary Information**</u>

Citric acid-mediated microwave-hydrothermal synthesis of mesoporous F-doped HAp nanorods from bio-waste for biocidal implant applications

Gopalu Karunakaran[1,*], Eun-Bum Cho[1,], Govindan Suresh Kumar[2], Evgeny Kolesnikov[3], Kattakgoundar Govindaraj Sudha[4], Kowsalya Mariyappan[4], Areum Han[5] and Shin Sik Choi[5,6]**

[1] Institute for Applied Chemistry, Department of Fine Chemistry, Seoul National University of Science and Technology (Seoul Tech), Gongneung-ro 232, Nowon-gu, Seoul, 01811, Republic of Korea, (G.K.) karunakarang5@seoultech.ac.kr, (E.-B. Cho) echo@seoultech.ac.kr
[2] Department of Physics, K.S. Rangasamy College of Arts and Science (Autonomous), Tiruchengode 637 215, Tamil Nadu, India, (G.S. Kumar.) gsureshkumar1986@gmail.com
[3] Department of Functional Nanosystems and High-Temperature Materials, National University of Science and Technology "MISiS," Leninskiy Pr. 4, Moscow 119049, Russia, (E.K.) kea.misis@gmail.com
[4] Department of Biotechnology, K.S. Rangasamy College of Arts and Science (Autonomous), Tiruchengode 637 215, Tamil Nadu, India, (K.G.S.) sudharhi@gmail.com, (K.M.) kowsalyamariyappan23@gmail.com
[5] Department of Food and Nutrition, Myongji University, Myongji-ro 116, Cheoin-gu, Yongin 17058, Republic of Korea (A.H.) gks0407@naver.com, (S.S. Choi) sschoi@mju.ac.kr
[6] Department of Energy Science and Technology, Myongji University, Myongji-ro 116, Cheoin-gu, Yongin 17058, Republic of Korea, (S.S. Choi) sschoi@mju.ac.kr

* Correspondence: (G.K.) karunakarang5@seoultech.ac.kr;
** Correspondence: (E.-B. Cho) echo@seoultech.ac.kr

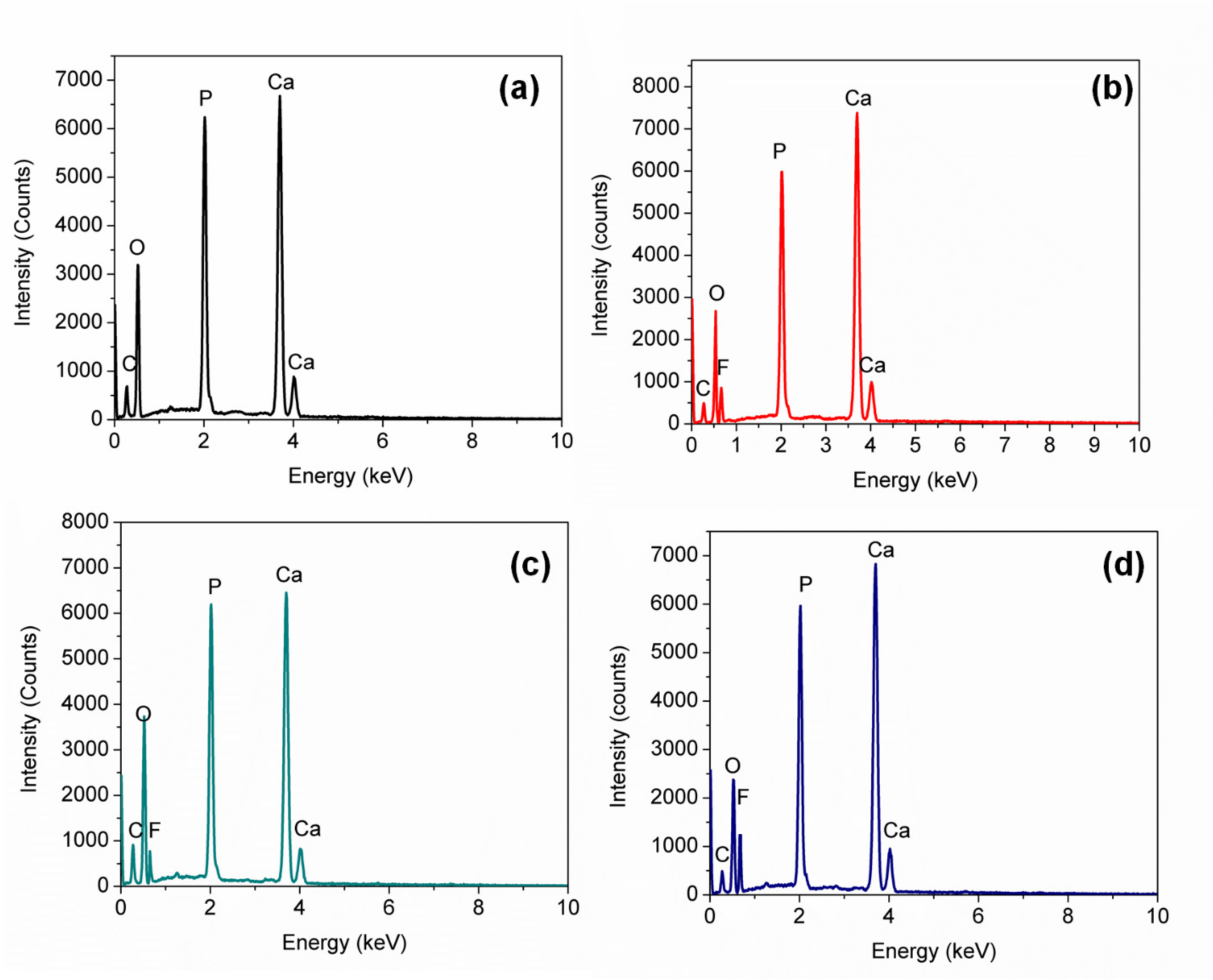

Figure S1. EDX spectra of (a) pure HAp, (b) FHAp-1, (c) FHAp-2 and (d) FHAp-3 samples.
